# Quaternized Cellulose Hydrogels as Sorbent Materials and Pickering Emulsion Stabilizing Agents

**DOI:** 10.3390/ma9080645

**Published:** 2016-07-30

**Authors:** Inimfon A. Udoetok, Lee D. Wilson, John V. Headley

**Affiliations:** 1 Department of Chemistry, University of Saskatchewan, 110 Science Place, Saskatoon, SK S7N 5C9, Canada; Inimfon.udoetok@usask.ca; 2 Water Science and Technology Directorate, Environment and Climate Change Canada, 11 Innovation Boulevard, Saskatoon, SK S7N 3H5, Canada; john.headley@canada.ca

**Keywords:** cellulose, hydrogel, sorption, cross-linking, quaternization, hydrophile–lipophile balance, cooperative interactions

## Abstract

Quaternized (QC) and cross-linked/quaternized (CQC) cellulose hydrogels were prepared by cross-linking native cellulose with epichlorohydrin (ECH), with subsequent grafting of glycidyl trimethyl ammonium chloride (GTMAC). Materials characterization via carbon, hydrogen and nitrogen (CHN) analysis, thermogravimetric analysis (TGA), and Fourier transform infrared (FTIR)/^13^C solid state NMR spectroscopy provided supportive evidence of the hydrogel synthesis. Enhanced thermal stability of the hydrogels was observed relative to native cellulose. Colloidal stability of octanol and water mixtures revealed that QC induces greater stabilization over CQC, as evidenced by the formation of a hexane–water Pickering emulsion system. Equilibrium sorption studies with naphthenates from oil sands process water (OSPW) and 2-naphthoxy acetic acid (NAA) in aqueous solution revealed that CQC possess higher affinity relative to QC with the naphthenates. According to the Langmuir isotherm model, the sorption capacity of CQC for OSPW naphthenates was 33.0 mg/g and NAA was 69.5 mg/g. CQC displays similar affinity for the various OSPW naphthenate component species in aqueous solution. Kinetic uptake of NAA at variable temperature, pH and adsorbent dosage showed that increased temperature favoured the uptake process at 303 K, where Q_m_ = 76.7 mg/g. Solution conditions at pH 3 or 9 had a minor effect on the sorption process, while equilibrium was achieved in a shorter time at lower dosage (ca. three-fold lower) of hydrogel (100 mg vs. 30 mg). The estimated activation parameters are based on temperature dependent rate constants, k_1_, which reveal contributions from enthalpy-driven electrostatic interactions. The kinetic results indicate an ion-based associative sorption mechanism. This study contributes to a greater understanding of the adsorption and physicochemical properties of cellulose-based hydrogels.

## 1. Introduction

Hydrogels are three-dimensional macromolecular polymer networks capable of absorbing and retaining significant water content due to its hydrophilic nature and network structure [1,2,3]. Hydrogels are usually prepared by various methods such as chemical cross-linking [4] and physical entanglement [5]. Biopolymer hydrogels derived from polysaccharides are very promising as carrier systems for drug delivery, stabilizers for Pickering emulsions, and adsorbents for pollutant removal. Biopolymer hydrogels are advantageous due to their abundance, low toxicity, biocompatibility, biodegradability, and tunable functionality [3,6,7,8]. Cellulose is an abundant and renewable biomaterial, white coloration, odourless, and nontoxic in nature. Cellulose also has unique properties such as mechanical strength, biocompatibility, hydrophilicity, relative thermal stability and tunable functionality [6]. The superior mechanical strength of cellulose over other polysaccharides with similar chemical structure and functionality relate to the strong versatile hydrogen bonding within and between biopolymer strands. This structural arrangement allows for segregation of hydrophobic and hydrophilic regions of the polymer to interact favourably with other components [9,10,11].

Cross-linking of cellulose-based hydrogels was reported using insoluble cellulose derivatives with bifunctional linkers such as epichlorohydrin [12], epichlorohydrin with aqueous ammonia [13], and divinylsulfone (DVS) [14]. As well, water soluble forms of cellulose containing quaternary ammonium salt derivatives [15,16] have also been explored. Previous reports [12,17] indicate that the point of zero charge (PZC) of cellulose-based hydrogels range from 6 to 8; thus, affording suitable uptake properties of both anionic and cationic adsorbates according to the pH conditions. However, these hydrogels are used for the sorption of anions such as naphthenic acid fraction components (NAFCs) found in oil sands process water (OSPW). At pH conditions above the zeta potential of the hydrogel, adsorptive uptake is limited due to electrostatic repulsion between the negatively charged hydrogel surface and the ionized naphthenates. However, the presence of hydrophilic moieties on the hydrogel surface may enhance the uptake affinity with amphiphilic naphthenate species, according to the “law of matching water affinities” [18]. OSPW is reported to display toxicity [19,20,21,22,23,24] due to the water soluble organic fraction that contains the major persistent toxic constituent, referred to as the NAFCs. NAFCs exhibit chemical stability, surfactant-like properties, persistence in the environment, non-volatility and high viscosity, especially at pH values above the pK_a_ of NAFCs. The growth in the oil sands extraction industry and the zero-discharge policy of the Government of Canada indicate the need for the development of low cost and sustainable technology for the treatment of the process-affected water. Biopolymer sorbents and their modified hydrogel forms may serve to address this need by subsequent reclamation of the vast area of land used for the storage of these tailings and process-affected water [25,26].

One approach to improve the affinity of cellulose hydrogels for contaminants like NAFCs at pH values above their point of zero charge (PZC) is to cross-link with bifunctional units such as epichlorohydrin before further grafting with quaternary ammonium salts such as glycidyl trimethyl ammonium chloride (GTMAC). Cross-linking with epichlorohydrin may enhance the surface area, porosity and hydrophobic character of the hydrogel [12], while grafting with quaternary ammonium salts will alter the hydrophile–lipophile balance (HLB) of the system. The shift of the PZC to higher pH values through the introduction of the quaternary ammonium groups affords charge stabilization of the hydrogel over a wider range of pH conditions [27]. 

The aim of this research was focused on understanding the role of cross-linking of cellulose with epichlorohydrin and surface modification via grafting with NH_4_OH and GTMAC, along with a study of the uptake properties of hydrogel materials with NAFCs. This research will contribute to the development of low cost and sustainable materials for the treatment of tailing ponds in several ways: (*i*) to provide a greater understanding of the chemistry of cellulose hydrogels by studying the effect of cooperative interactions for hydrogel/adsorbate systems; (*ii*) to study the effect of cellulose cross-linking before the introduction of quaternary ammonium ions on the adsorption properties of such hydrogel systems; and (*iii*) to evaluate the utility of cellulose hydrogels as Pickering emulsion stabilizers and as sorbent materials for the uptake of NAFCs.

## 2. Results and Discussion

### 2.1. Characterization of Cellulose Hydrogels

#### 2.1.1. FTIR Studies

**Figure 1:**
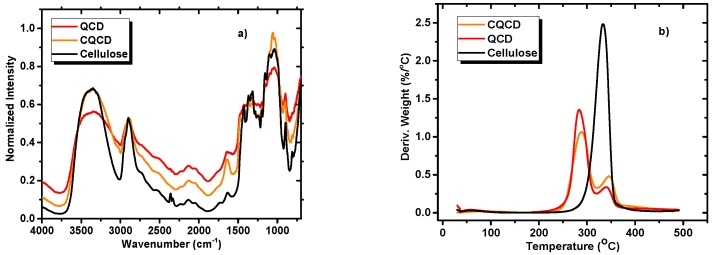
(**a**) FTIR spectra; and (**b**) DTG plot of cellulose and the hydrogels.

The Fourier transform infrared (FTIR) spectra of the cellulose hydrogels and native cellulose are displayed in Figure 1a. The salient features of the spectra include a broad band (~3000–3600 cm^−1^) attributed to intermolecular bonded OH and NH groups, C–H stretching (~2800–3000 cm^−1^), O–H and C–H bending (~1400–1300 cm^−1^), and C–O–H and C–O–C asymmetric stretching (~1000–1200 cm^−1^), in agreement with a study on cross-linked cellulose [12]. The spectra reveal differences between native cellulose and the hydrogels, where the introduction of methylene, methyl and quaternary ammonium groups occur due to cross-linking and/or quaternization reactions, in agreement with reported studies [28,29]. The different features include IR bands at 1485 cm^−1^, ~1000–1200 cm^−1^ and ~2934 cm^−1^ which display greater intensity relative to native cellulose, along with attenuation of the bands at ~1300–1400 cm^−1^. The new IR bands relate to the methyl groups from the quaternary ammonium ion, ether linkage from cross-linking with epichlorohydrin, as well as the methylene groups due to cross-linking and/or quaternization reactions, in agreement with other reports [30,31]. An increased intensity and broadening of the band at ~1640 cm^−1^ relates to adsorbed water molecules and the C–N band at ~1450 cm^−1^ for the hydrogels provide further evidence for the introduction of quaternary ammonium groups onto the surface sites of cellulose [17,30].

#### 2.1.2. Thermogravimetry Studies

The thermal stability of native cellulose and the hydrogels are inferred from the DTG plot of derivative weight (%/°C) vs. temperature (°C) in Figure 1b. The thermogravimetric analysis (TGA) results show a single thermal event for cellulose relative to two thermal events for the hydrogels, in agreement with an independent report [32]. The maximum decomposition temperature for pristine cellulose occurs at ~320 °C; whereas, the hydrogels display maximum decomposition at ~260 °C and ~360 °C. The two thermal events for the hydrogels differ compared to cellulose, where a single thermal event is shown. The TGA results of the hydrogels relate to cross-linking and/or quaternization of cellulose, in agreement with the FTIR band at 1485 cm^−1^ in Figure 1a. The lower temperature event (~260 °C) for the hydrogels correspond to the cross-linker and quaternary ammonium groups; whereas, the event at ~360 °C relates to the decomposition of cellulose [32]. The DTG profiles also reveal that the intensity of the thermal event due to the decomposition of the cross-linker and quaternary ammonium groups of the hydrogel differ from the profile of cellulose. The foregoing provides support that cellulose is modified via cross-linking and quaternization. The profile also reveals that the cross-linked and quaternized hydrogel (CQC) has greater thermal stability over the quaternized hydrogel (QC). This greater stability of the cross-linked and quaternized hydrogel may relate to cross-linking effects, as noted for cellulose in another study [12].

#### 2.1.3. Carbon, Hydrogen and Nitrogen (CHN) Composition of Hydrogels

The CHN results of the hydrogel are compared with cellulose in Table 1. The hydrogel contains greater C, H, and N content than unmodified cellulose. The presence of nitrogen in the hydrogels confirms the grafting of quaternary ammonium groups, in agreement with the above FTIR and DTG results, along with a related study [33]. The results reveal that CQC contains more nitrogen than QC and may be a consequence of the cross-linking of cellulose with ECH and grafting with aqueous ammonia before the quaternization process. The effect corresponds with a related study for the cross-linking of hydroxypropyl cellulose with ECH and aqueous ammonia [13].

#### 2.1.4. Hydrophile–Lipophile Balance (HLB) of Cellulose Hydrogels

**Figure 2:**
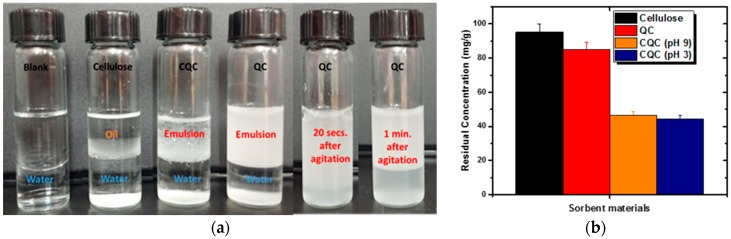
(**a**) Stabilization of oil/water emulsions by cellulose and hydrogels; and (**b**) uptake of NAA by cellulose and the hydrogels at pH 3 and 9 at 293 K.

The relative HLB of the cellulose materials was estimated by proxy, according to results obtained for solvent swelling in octanol and water (cf. Table 1 and Figure 2). The swelling results reveal that QC without cross-linking displayed greater swelling in water and octanol relative to its cross-linked form (CQC). The ratio of the swelling in octanol and water reveal that QC has measureable differences in HLB over CQC (CQC = 0.720; QC = 0.625). The HLB results are affirmed by QC which displays the greatest stabilization of a hexane in water Pickering emulsion system (cf. Figure 2a), exceeding four weeks. The colloidal stabilization of the emulsion by QC may be due to its greater wetting properties and ability to reduce the interfacial energy by adsorption at the oil/water interface [34,35]. The foregoing also reveals that CQC is a more hydrophobic hydrogel as evidenced by its HLB value, in agreement with the greater sorptive uptake of 2-naphthoxy acetic acid (NAA) (cf. Figure 2b), an amphiphilic adsorbate.

#### 2.1.5. ^13^C NMR Studies of Cellulose Hydrogels

**Figure 3:**
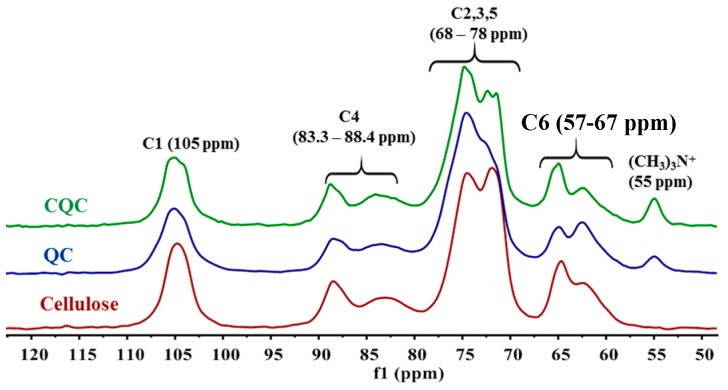
^13^C CP-MAS solids NMR spectra of cellulose, QC and CQC obtained at 125.8 MHz with MAS at 10 kHz and 293 K.

The ^13^C solids NMR spectra of native cellulose and the hydrogels are presented in Figure 3. Unmodified cellulose display the following ^13^C resonances: C1 (105 ppm), C2/C3/C5 (68–78 ppm), C4 (88.4 and 83.3 ppm), and C6 (57–67 ppm), in agreement with a previous report [36]. The ^13^C spectra of the hydrogels possess features that resemble native cellulose, in agreement with the component nature of the cellulose biopolymer structural units. The hydrogels reveal new ^13^C NMR resonance lines, band broadening, and chemical shift variations. These effects are absent in the spectra of cellulose, in accordance with the DTG and FTIR results above. For example, new resonance lines at ~55.0 ppm for (CH_3_)_3_N^+^ and ~70–77 ppm relate to CH_2_ groups from ECH and GTMAC, respectively, in agreement with other reports [37,38]. The resonance lines for ECH and GTMAC may overlap with the spectral features (cf. ~62–65 ppm and ~68–78 ppm) of native cellulose, in accordance with the line broadening related to quaternization [38]. The shoulder at ~103 ppm may be due to C3 which bears substituted or non-substituted hydroxyl groups [39]. The difference between CQC and QC in the ^13^C NMR spectra relate to the well resolved and increased spectral intensity of the lines ~68–78 ppm from ECH and GTMAC, related to their similar chemical environments. QC was synthesized via grafting cellulose with GTMAC without cross-linking. The ^13^C NMR results provide support that cross-linking occurs between cellulose and ECH with quaternization due to GTMAC, in agreement with the above DTG results.

### 2.2. Sorption Studies

#### 2.2.1. Comparative Uptake of Single Component Carboxylate Anion

Sorption results for the uptake affinity of cellulose and the hydrogels with NAA in aqueous solution are shown in Figure 2b. The results reveal that CQC displays greater affinity for the uptake of NAA relative to cellulose and QC. The greater affinity of CQC with NAA likely relate to the *surfactant-like* properties of NAA, in accordance with the HLB results presented above for CQC. Cross-linking of cellulose with ECH results in reduced water solubility; whereas, grafting with GTMAC further enhances the hydrophile character due to the introduction of quaternary ammonium groups. Thus, the uptake of NAA by CQC is a result of cooperative interactions. The uptake results in Figure 2b shows that pH has a minor effect on the equilibrium uptake of NAA by CQC due to the grafting of the quaternary ammonium ions onto cellulose. Previous reports on the uptake properties [12] of anions with cellulose materials indicate that low anion affinity occurs at alkaline pH as a result of the negative zeta-potential on the sorbent surface [40]. The introduction of quaternary ammonium groups offset the low anion affinity in the case of unmodified cellulose through the introduction of a positive zeta-potential, as for the case of QC or CQC.

#### 2.2.2. Equilibrium Studies of Single Component Carboxylate Anion (NAA) and OSPW Naphthenates

**Scheme 1:**
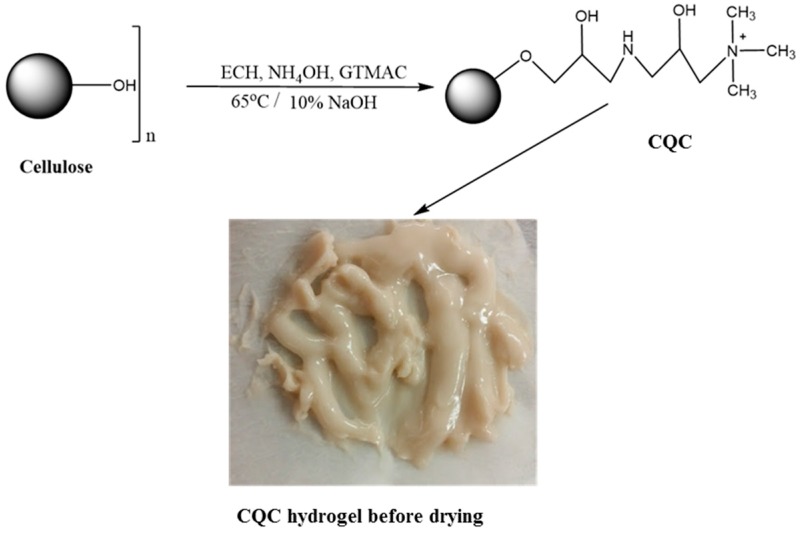
Synthetic scheme for the preparation of cross-linked/quaternized CQC.

**Figure 4:**
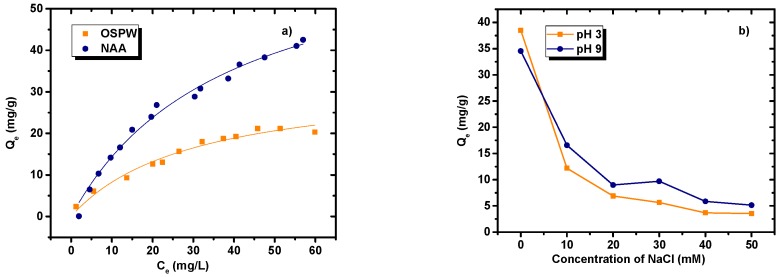
(**a**) Sorption isotherm of CQC with NAA at pH 9 and OSPW at pH 10.5 and 293 K; and (**b**) effect of ion concentration on the sorption isotherm of NAA by CQC at pH 3 and 9 and 293 K.

The uptake isotherms of the sorbents with NAA and OSPW naphthenates are presented as plots of Q_e_ versus C_e_ in Figure 4a. The isotherms display a non-linear increase for Q_e_ as C_e_ increases. The Langmuir model provides a reasonable “best-fit” to the experimental results and reveal that the sorbent surface has homogenous sorption sites (cf. Table 2). The proposed structure of CQC in Scheme 1 agrees with the ^13^C solids NMR and FTIR spectral results, where two possible sorption sites exist in such sorbent materials. These include the quaternary ammonium sites and the hydrophobic domains of cellulose even though the isotherm results are described by the Langmuir model. Evidence of a singular type of sorption site for these materials can be understood on the basis of a large offset in the binding affinity of each respective site. The uptake of NAA and OSPW naphthenates by CQC is likely dominated by the ion–ion electrostatic attractions, aided by cooperative hydrophobic effects. The Langmuir isotherm data shows that CQC displayed a two-fold greater uptake of NAA (Q_m_ = 69.5 mg/g) over OSPW naphthenates (Q_m_ = 33.0 mg/g). The variable uptake of NAA by CQC may be due to the mixed composition of OSPW, where certain congeners have reduced uptake due to steric effects or variable HLB that limit the binding with CQC. The variable lipophilicity profile of OSPW relates to the z-value of the naphthenates, where the z-value denotes the “hydrogen deficiency”, as reported elsewhere [41,42,43,44,45]. Naphthenates with z-values less than 2 in the OSPW display enhanced binding affinity with CQC, as follows: OSPW (0.0333 L/mg) and NAA (0.234 L/mg). The hydrogel reported herein has favourable uptake relative to activated carbon (AC) and nickel-based alumina (Ni-Al_2_O_3_), 20 mg/g, and chemically treated AC, 35 mg/g, for OSPW naphthenates [46,47].

#### 2.2.3. Effects of Ion Concentration on the Uptake of NAA

The concentration effect of NaCl on the sorption of NAA with CQC is presented in Figure 4b. The results reveal that an increase in the level of NaCl in the NAA solution results in reduced uptake of NAA with CQC, in agreement with other reports [46,48]. The uptake of NAA decreased from 38 mg/g to 5 mg/g at pH 3, and 35 mg/g to 6 mg/g at pH 9 as the level of NaCl increased to 50 mM. The attenuated uptake of NAA in the presence of electrolyte may be due to charge screening effects at the sorption sites between the carboxylate anions and the quaternary ammonium groups, in agreement with enhanced ion–ion binding contributions, as outlined above.

#### 2.2.4. Sorption of OSPW Naphthenates by Cellulose Hydrogels

**Figure 5:**
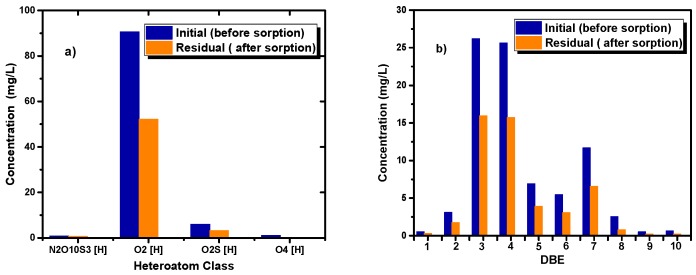
(**a**) Electrospray ionization high resolution mass spectrometry (ESI-HRMS) speciation profile of OSPW; and (**b**) double bond equivalents (DBE) distribution of OSPW as a function of normalized concentration for the O_2_ species before and after sorption with CQC at pH 10.5 and 293 K.

The ESI-HRMS speciation profile of OSPW before and after sorption with CQC is shown in Figure 5a,b. Figure 5a displays the class distribution plots for OSPW and show that O_2_H species are the most prominent; whereas, the O_2_S species are of secondary abundance. The results demonstrate a significant attenuation of the concentration of the O_2_H species after sorption with the CQC hydrogel. The foregoing shows that the CQC hydrogel has favourable uptake affinity and removal of these OSPW fractions at equilibrium. 

Figure 5b presents a distribution profile of double bond equivalent (DBE) species in OSPW as a function of the normalized concentration of O_2_ species. The DBE accounts for the hydrogen deficiency due to ring formation or double bonds of the naphthenate systems [49]. The results show that the O_2_H species are comprised of naphthenates with DBE values in the range of 1 to 10. In addition, the more prominent species in the OSPW mixture have DBE values between 2 and 8. The uptake results show that CQC has little or no selectivity toward the carboxylate anion species in the mixture, in agreement with the electrostatic driven binding for such hydrogel/OSPW systems. The removal efficiency ranged from 38% (DBE = 4) to 69% (DBE = 8), while Mohamed et al. [50] reported an increase in removal efficiency with increasing DBE due to hydrophobic effects. Udoetok et al. [12] reported greater uptake of model naphthenates as the number of rings increased for cellulose and its cross-linked forms. The foregoing shows that CQC has appreciable fractionation efficacy for OSPW naphthenates that may relate to cooperative electrostatic and hydrophobic effects, as noted above.

### 2.3. Kinetic Uptake Studies

#### 2.3.1. Effects of Temperature and Sorbent Dosage

**Figure 6:**
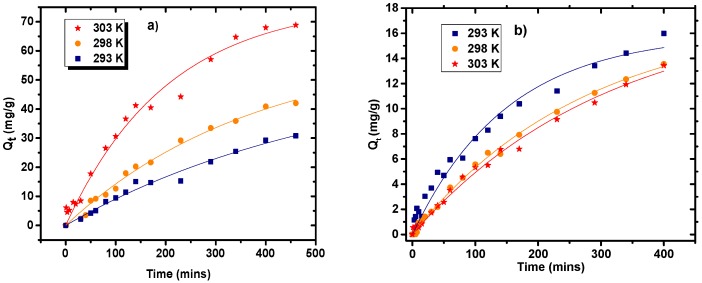
Kinetic uptake profile of NAA: (**a**) low dosage (30 mg); and (**b**) high dosage (100 mg) of CQC at pH 9 and 293 K, 298 K and 303 K. The fitted lines correspond to the PFO model.

Figure 6 illustrates the effects of temperature on the kinetic uptake of NAA from aqueous solution by CQC via the one-pot kinetic system. The uptake profile is well described by the pseudo-first order (PFO) kinetic model, in agreement with a single type of sorption site. The results show an increase in Q_t_ values with increasing time, where saturation occurs at t > 400 s. In Figure 6a, the kinetic profile reveals that Q_e_ (mg/g) and the PFO rate constant, k_1_ (s^−1^) increase with temperature at a low dosage (30 mg) of CQC (cf. Table 3), where dynamic equilibrium is reached after 460 min. At higher hydrogel dosage (100 mg) in Figure 6b, the rate becomes attenuated as temperature increases, due to the fact that the monolayer saturation of the biopolymer was not met after 400 min. A comparison of the Q_e_ values for each adsorbent dosage (cf. Table 4) reveal that uptake of CQC (30 mg dosage) at 303 K was higher relative to uptake for the hydrogel (100 mg dosage), in support of the claim that monolayer saturation was not achieved after 400 s. Figure 6b shows that the uptake profile at 303 K converged with the results at 298 K, supporting the claim that longer time is required to achieve dynamic equilibrium. The increase of Q_e_ and k_1_ values as temperature increases indicate that higher temperature provides the required energy to activate the adsorption process, in agreement with other results [51]. 

#### 2.3.2. Effects of pH

**Figure 7:**
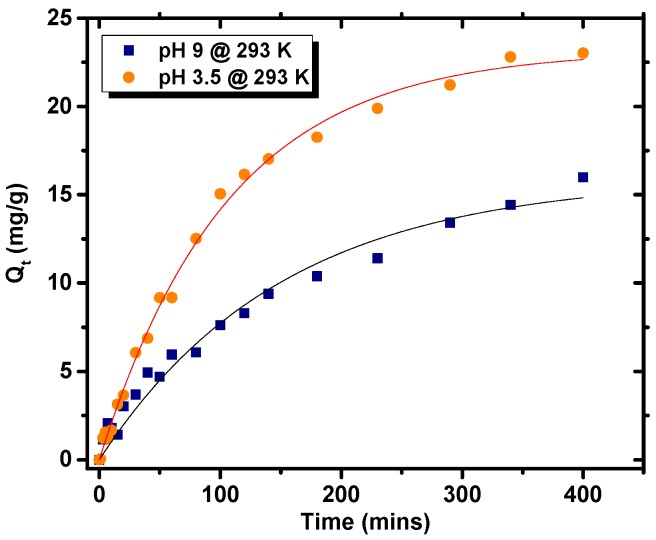
Kinetic uptake profile of NAA at 293 K with a 0.833 mg/mL dosage of CQC at pH 9 and 3, where the fitted lines correspond to the PFO model.

A study of pH effects on the uptake kinetics of NAA by CQC hydrogel is shown in Figure 7. The results show an increase for Q_t_ as time increases that agree with general effects of temperature and sorbent dosage. The kinetic uptake results at pH 3 (Q_e_) and the PFO rate constant (k_1_) are greater relative to conditions at pH 9 (cf. Table 3). These results do not agree with the comparative equilibrium uptake of NAA by cellulose and the hydrogels at pH 3 and 9. The slight disparity in the uptake capacity between the equilibrium and kinetic studies is related to the earlier claim that 400 min is insufficient for achieving isotherm saturation, and the faster rate of the uptake at pH 3. The greater PFO rate constant obtained for the kinetic uptake at pH 3 is related to the reduced role of counter ion binding with the quaternary ammonium ions at acidic pH relative to pH 9, where greater charge screening effects are anticipated as the level of OH^-^ increases. 

#### 2.3.3. Activation Parameters

**Figure 8:**
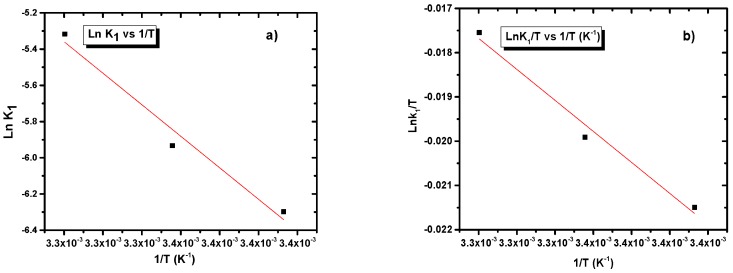
(**a**) Plot of ln k_1_ versus 1/T for the determination of activation energy (∆E_a_); and (**b**) plot of ln k_1_ /T versus 1/T for the determination of activation parameters of adsorption for the CQC–NAA system.

The calculated activation parameters such as the change in Gibbs energy (*ΔG*), enthalpy change (*ΔH*), entropy change (*ΔS*), and activation energy (*ΔE_a_*), were based on the trend in PSO rate constants (k_1_) with temperature. The activation parameters were calculated from a plot of ln k_1_/T versus 1/T (cf. Figure 8b) by the Eyring equation (Equation (9)). The positive value of ΔG* (cf. Table 4) at variable temperature shows a decrease with increasing temperature, indicating that the sorption process is exergonic. The endothermic nature of ΔH* of the sorption process is anticipated for electrostatic driven processes. Based on the ΔS* values, an associative sorption mechanism is inferred which proceeds via the formation of an activated complex for the hydrogel system [52]. The Δ*E_a_* value in Table 4 derived from the plot of ln k_1_ versus 1/T (cf. Figure 8a) by the Arrhenius equation (Equation (11)) is 72 kJ/mol. The result agrees with estimates of *E_a_* for other ion exchange systems reported elsewhere [53]. The foregoing along with the Langmuir-based description for the adsorption process support the claim that electrostatic interactions are predominant with evidence of cooperative hydrophobic effects for the uptake of NAA by CQC.

## 3. Materials and Methods

### 3.1. Materials

Cellulose (medium fibre from cotton linters), sodium hydroxide, aqueous ammonia, sodium chloride, 99% epichlorohydrin (ECH) glycidyl trimethyl ammonium chloride (GTMAC), HCl, and ACS grade acetone and octanol were obtained from Sigma Aldrich (St. Louis, MO, USA). All materials were used as received without further purification.

### 3.2. Synthesis of Cellulose Hydrogels

Synthesis of a cellulose hydrogel (CQC) was carried out using 2 g of bulk cellulose with heating in 15 mL of 10% NaOH at 65 °C for 3 h. One millilitre of ECH followed by 1 mL of NH_4_OH was added to the NaOH-cellulose mixture in a drop-wise manner over a one-minute period, where the reaction mixture was stirred at 65 °C for ca. 16 h. The resulting reaction mixture was neutralized using 6 M HCl and the product separated from the supernatant via vacuum filtration, followed by washing with cold Millipore water and ACS grade acetone. The resulting product was added to another round bottom flask and heated in 15 mL of 10% NaOH 65 °C for 1 h followed by the drop-wise addition of 10 mL glycidyl trimethyl ammonium chloride (GTMAC). The reaction mixture was allowed to stir for at least 6 h before separation of the final products via vacuum filtration, followed by washing with cold Millipore water and ACS grade acetone with drying at 60 °C. Soxhlet extraction for 24 h with HPLC grade acetone was used to remove unreacted/excess reagents, followed by drying in a vacuum oven at 60 °C for 12 h. The resulting polymer was ground in a mortar and pestle and sieved with a 40-mesh sieve. Another hydrogel (QC) was also synthesized via the same method but without the ECH and NH_4_OH for the cross-linking step.

### 3.3. Characterization

#### 3.3.1. Fourier Transform Infrared (FTIR) Spectroscopy 

The FTIR spectra of the hydrogels and cellulose were obtained using A Bio-RAD FTS-40 IR spectrophotometer (Bio-Rad Laboratories, Inc., Philadelphia, PA, USA). Dry and powdered samples were mixed with pure spectroscopic grade KBr in a weight ratio of 1:10 with grinding in a small mortar and pestle. The DRIFT (Diffuse Reflectance Infrared Fourier Transform) spectra were obtained in reflectance mode at room temperature with a resolution of 4 cm^−1^ over the 400–4000 cm^−1^ spectral range. Multiple scans were recorded and corrected relative to a background of pure KBr.

#### 3.3.2. Thermal Gravimetric Analysis (TGA)

Thermal stability of the hydrogels and cellulose were determined using a TA Instruments Q50 TGA system (New Castle, DE, USA) operated with a heating rate of 5 °C min^−1^ up to 500 °C using nitrogen as the carrier gas. The results obtained are shown as first derivative (DTG) plots of weight with temperature (%/°C) against temperature (°C).

#### 3.3.3. Carbon, Hydrogen and Nitrogen (CHN) Analyses

The C, H, and N composition of the hydrogels and cellulose were obtained using a Perkin Elmer 2400 CHN Elemental Analyzer (PerkinElmer, Inc., Waltham, MA, USA) with the following instrument conditions: combustion oven temperature (above 925 °C) and reduction oven temperature (above 640 °C). The instrument was purged with a mixture of pure oxygen and helium gas, where acetanilide was used as the calibration standard. Elemental analyses were obtained in triplicate with an estimated precision of ±0.3%.

#### 3.3.4. Hydrophile–Lipophile Balance (HLB)

The hydrophile–lipophile balance (HLB) of the hydrogels was estimated via swelling in Millipore water and octanol, respectively. This was done by shaking approximately 30 mg of the materials in 15 mL of Millipore water and octanol in a horizontal shaker for ~48 h. The weights of swollen hydrogels (w_s_) were determined after tamping dry with filter paper. The dry weights (w_d_) were obtained after drying in an oven at 65 °C to a constant mass. The swelling ratio was calculated for each neat solvent system using Equation (1):

(1) $$Sw\left(\%\right)=\frac{\mathrm{Ws}-\mathrm{Wd}}{Wd}\times 100$$

#### 3.3.5. Solid State ^13^C NMR Spectroscopy

A Bruker AVANCE III HD spectrometer (Bruker Bio Spin Corp., Billerica, MA, USA) furnished with a 4 mm DOTY CP-MAS (cross polarization with magic angle spinning) solids probe operating at 125.8 MHz (^1^H frequency at 500.2 MHz) was use to acquire the ^13^C solids NMR spectra of the hydrogels and cellulose. The experimental conditions were as follows: spinning speed of 10 kHz, a ^1^H 90° pulse of 3.5 µs, a contact time of 0.75 ms, with a ramp pulse on the ^1^H channel. Others are MAS rate of 10 kHz, a ^13^C 90° pulse of 3.15 µs and a 25 kHz SPINAL-64 ^1^H decoupling during acquisition. For different samples, 600–5000 scans were accumulated, with a recycle delay of 2 s. All experiments were recorded using 71 kHz SPINAL-64 decoupling during acquisition. Chemical shifts were referenced to adamantane at 38.48 ppm (low field signal). 

### 3.4. Sorption Studies

#### 3.4.1. Sorption of OSPW Naphthenates and Single Component Carboxylate Ions

A 100-mL stock solution at 100 ppm was prepared for the OSPW and 150 ppm for 2-naphthoxy acetic acid (NAA), respectively. An appropriate amount of NAA was dissolved in an aqueous NH_3_ solution with sonication and further stirring until the resulting solution was clear, while 2800 ppm OSPW was diluted using Millipore water and stirred overnight. Solutions with variable concentration (1–150 ppm) were prepared by dilution of the stock with Millipore water.

Fixed amounts (10 mg) of the hydrogels were mixed with 5 mL of NAA and OSPW solutions in 2 dram vials at variable concentration. The mixtures were equilibrated at room temperature on a horizontal shaker table for 24 h. The initial concentration (C_o_) and residual concentration at equilibrium (C_e_) of OSPW naphthenates and NAA were determined using an electrospray ionization high resolution mass spectrometer and a double beam spectrophotometer (CARY 100, Varian, Mulgrave, Australia) at room temperature (293 ± 0.5 K). The samples were centrifuged prior to ESI-HRMS and UV-vis spectroscopy analyses. Uptake of NAA and NAFCs in OSPW was determined from the difference between C_o_ and C_e_ values described by Equation (2).

(2) $$Q_{e}=\frac{(C_{o}-C_{e})\times V}{m}$$

Q_e_ is the quantity of adsorbate uptake in the solid phase at equilibrium (mg·g^−1^); C_o_ is initial concentration of adsorbate (mg·L^−1^) in solution; C_e_ is concentration of adsorbate at equilibrium (mg·L^−1^) in solution; V is volume of adsorbate solution; and m is the mass (g) of sorbent.

#### 3.4.2. Electrospray Ionization Mass Spectrometric (ESI-HRMS) Quantification

The residual (C_e_) and initial (C_o_) concentration of the NAFCs was estimated using a ThermoScientific LTQ Orbitrap Elite Electrospray ionization high resolution mass spectrometer (ESI-HRMS) (Thermo Fisher Scientific Inc., Waltham, MA, USA). The resolution setting of the spectrometer was 30,000 while a full-scan mass spectrum was collected between *m/z* 100 and 600. Quantification of samples was achieved by extracting the mass range of the analyte of interest. The electrospray ionization (ESI) interface was set to negative-ion mode. Mass spectrometer conditions were optimized by the transmission of *m/z* 112.98563. The heated ESI interface (HESI) parameters were as follows: source heater temperature (53 °C); spray voltage (2.86 kV); capillary temperature (275 °C); sheath gas flow rate (25 L·h^−1^); auxiliary gas flow rate (5 L·h^−1^); and spray current (5.25 μA).

#### 3.4.3. Sorption Isotherms and Modeling

Sorption isotherms were obtained by plotting Q_e_ vs. C_e_ (cf. Equation (2)). The isotherms were subsequently analyzed using Langmuir [54] Freundlich [55] and the Sips [56] models (cf. Equation (3)–(5)). The “best fit” for the data was obtained by minimizing the SSE (cf. Equation (6)) for all data across the range of conditions. Q_ei_ is the experimental value, Q_ef_ is the calculated value from data fitting and N is the number of Q_e_ data points.

(3) $$Q_{e}=\frac{K_{L}Q_{m}C_{e}}{1+K_{L}C_{e}}$$

(4) $$Q_{e}=K_{F}{C_{e}}^{1/n_{f}}$$

(5) $$Q_{e}=\frac{Q_{m}{\left(K_{S}C_{e}\right)}^{n_{s}}}{1+{\left(K_{S}C_{e}\right)}^{n_{s}}}$$

(6) $$\mathrm{SSE}=\sqrt{\frac{{\left(Q_{\mathrm{ei}}-Q_{\mathrm{ef}}\right)}^{2}}{N}}$$

### 3.5. Kinetic and Thermodynamic Studies

#### 3.5.1. Kinetic Studies

Kinetic studies were carried out using a one pot method [57] as follows: variable amounts (~100 mg or 30 mg) of the hydrogel were added to a folded Whatman filter paper (55 mm diameter), where both ends were clamped after encasing the polymer. The clamped filter paper containing the sample was immersed in a fixed volume (120 mL) of a 150-ppm NAA solution. Three millilitres of the NAA solution were sampled after designated time intervals. The residual concentration of the aliquots was determined using a double beam spectrophotometer (Varian CARY 100) at 293 ± 0.5 K. Uptake of NAA at each sampling time interval (t) was estimated according to Equation (7), where C_o_ and C_t_ refer to the surrogate concentration at t = 0 and variable t.

(7) $$Q_{t}=\frac{(C_{o}-C_{t})\times V}{m}$$

Kinetic uptake isotherms were obtained by plotting Q_t_ vs. time according to the pseudo-first order (PFO kinetic model (cf. Equation (8)).

(8) $$Q_{t}=Q_{e}(1-e^{-k_{1}t})$$

#### 3.5.2. Thermodynamic Studies

The standard enthalpy of activation (ΔH*), entropy of activation (ΔS*), and the Gibbs energy of activation (ΔG*) in the adsorption process was calculated from a plot of ln k_1_/T versus 1/T according to the Eyring equation (Equation (9)),

(9) $$\frac{\ln\;k}{T}=\ln\frac{k_{B}}{h}+\frac{\Delta S^{*}}{R}-\frac{\Delta H^{*}}{\mathrm{RT}}$$

where k_1_ is the adsorption rate constant; k_B_ is the Boltzmann constant (1.3807 × 10^−23^ J·K^−1^); h is Planck’s constant (6.6261 × 10^−34^ J·s); R is the ideal gas constant (8.314 J·mol^−1^·K^−1^); and T is the temperature (K). The values of ΔH* and ΔS* were determined from the slope and intercept of a plot of ln k_1_/T versus 1/T. The values obtained were used to compute ΔG* from Equation (10) below:

ΔG* = ΔH* − TΔS*
(10)

The activation energy (E_a_) of the process was obtained by plotting *ln* k_1_ versus 1/T according to the Arrhenius Equation (11),

(11) $$\ln k_{1}=\ln A+\frac{E_{a}}{R}\left(\frac{1}{T}\right)$$

where *k_1_* is the rate constant; *A* is the pre-exponential factor; *E*_a_ is the activation energy; R is the gas constant; and *T* is the temperature (K). 

## 4. Conclusions

Quaternized cellulose hydrogels as cross-linked (CQC) and non-cross-linked forms (QC) were prepared. The grafting of GTMAC with cellulose results in the presence of quaternary ammonium groups, according to structural support via FTIR, CHN analysis, ^13^C NMR and TGA. The hydrogels possess enhanced network structure and variable hydrophobic character due to cross-linking and grafting effect. The TGA results reveal enhanced thermal stability of the hydrogels relative to cellulose, while solvent swelling studies in octanol and water indicate that QC has variable hydrophile–lipophile balance (HLB) relative to CQC. QC in hexane–water mixtures afford the formation of stable oil/water Pickering emulsions. The sorption of OSPW naphthenates and NAA by the hydrogels revealed that CQC has greater affinity over QC, where the uptake is favoured by cooperative hydrophobic and electrostatic interactions. CQC displays similar affinity for the component species of OSPW naphthenates, where the PFO model provides best fit for the experimental kinetic uptake results. The kinetic uptake process is favoured by increased temperature while pH has a minor effect. The activation parameters were derived from temperature dependence of the rate constants, k_1_, where an entropy driven sorption process appears to follow an associative ion-ion driven sorption mechanism. This study contributes to a greater understanding of the structure-function properties of cellulose hydrogels and their uptake properties with carboxylate anions. This study will catalyze further development of low-cost and versatile biopolymer materials for tunable Pickering emulsions, chemical fractionation, and diverse adsorption-based processes.

## Figures and Tables

**Table 1:** CHN content (%) and solvent swelling results of cellulose and its modified forms.

Material	% C	% H	% N	Swelling (Water)%	Swelling (Octanol)%	HLB *
Cellulose	41.0	6.27	NA	125	NA	NA
CQC	42.4	6.48	1.17	292	211	0.720
QC	42.8	7.43	0.80	415	260	0.625

* HLB is estimated by proxy as the ratio of swelling in octanol: water.

**Table 2:** Sorption isotherm parameters obtained from the Langmuir, Sips, and Freundlich models for CQC with OSPW naphthenates (pH 10.5) and NAA (pH 9.0) at 293 K.

Adsorbates	Isotherm Model	Parameters	Sorbent (CQC)
**OSPW**	Langmuir	**Q_m_ (mg·g^−1^)**	33.0
		**K_L_ (L·mg^−1^)**	0.0333
		**SSE**	0.921
	Sips	**Q_m_ (mg·g^−1^)**	36.9
		**K_S_ (L·mg^−1^)**	0.0370
		**n_s_**	0.904
		**SSE**	2.73
	Freundlich	**K_F_ (L·mg·g^−1^)**	2.63
		**1/n_f_**	1.89
		**SSE**	2.75 × 10^3^
**NAA**	Langmuir	**Q_m_ (mg·g^−1^)**	69.5
		**K_L_ (L·mg^−1^)**	0.0260
		**SSE**	0.994
	Sips	**Q_m_ (mg·g^−1^)**	60.5
		**K_S_ (L·mg^−1^)**	0.0234
		**n_s_**	1.12
		**SSE**	4.10
	Freundlich	**K_F_ (L·mg·g^−1^)**	3.47
		**1/n_f_**	2.09
		**SSE**	1.13 × 10^6^

**Table 3:** Pseudo-first order (PFO) kinetic uptake results of NAA by CQC at 293 K, 298 K and 303 K and pH 3 and 9.

Weight of Adsorbent	Temperature (K)	pH	Parameters		
			Q_m_ (mg/g)	K_1_ (S^−1^)	R^2^
30	293	9	54.2	0.00184	0.980
	298		61.3	0.00265	0.992
	303		76.7	0.00491	0.982
100	293		16.0	0.00666	0.974
	298		17.4	0.00634	0.997
	303		18.0	0.00319	0.992
	293	3	23.2	0.00943	0.996

**Table 4:** Thermodynamic parameters for the uptake of NAA by CQCD.

Temp (K)	∆E_a_ (kJ/mol)	Activation Parameters		
		∆H* (kJ/mol)	∆S* (J/Kmol)	∆G* (kJ/mol)
293	72.3	0.291	−196	57.9
298				58.9
303				59.8
